# Integrating Artificial Intelligence with Wearable Sensors for Advanced Health Monitoring and Diagnosis

**DOI:** 10.3390/bios16060344

**Published:** 2026-06-18

**Authors:** Dongyoun Kim, Syed Saad Ahmed, Amirhossein Amjad, Kwanghee Won, Xiaojun Xian

**Affiliations:** McComish Department of Electrical Engineering and Computer Science, Jerome J. Lohr College of Engineering, South Dakota State University, Brookings, SD 57007, USA; dongyoun.kim@jacks.sdstate.edu (D.K.); syed.ahmed@jacks.sdstate.edu (S.S.A.); amirhossein.amjad@jacks.sdstate.edu (A.A.)

**Keywords:** wearable sensors, artificial intelligence (AI), machine learning (ML), healthcare, diagnosis

## Abstract

Wearable healthcare technologies are transforming the healthcare landscape by enabling remote, real-time health data collection, supporting early diagnosis, personalizing treatment plans, and reducing healthcare costs and medical burdens. Central to these advancements are wearable sensors, which continuously capture physiological data such as heart rate, temperature, activity levels, and biomarker concentrations. However, the large volume and complexity of this data demand effective processing to extract meaningful medical insights. Artificial intelligence (AI) and machine learning (ML) have significantly enhanced the capabilities of wearable sensors by enabling advanced data analysis, pattern recognition, and predictive modeling. AI-enhanced wearable sensors can detect early signs of health issues, such as heart attacks, chronic diseases, and mental health conditions like stress, often before clinical symptoms become apparent. This review examines the integration of AI/ML models with wearable sensors across physical activity recognition, stress assessment, cardiovascular monitoring, personal exposure monitoring, and sweat biomarker detection. Unlike prior application-centered reviews, we emphasize methodological and translational evaluation by comparing task formulations, sensing modalities, dataset scale, validation protocols, performance metrics, and deployment constraints across domains. We further discuss advanced architectures, multimodal fusion, explainable AI, edge deployment, privacy and regulatory considerations, and the translational gap between research prototypes and clinically deployable wearable AI systems.

## 1. Introduction

Wearable sensors are electronic sensing systems designed to continuously monitor physical, biochemical, or behavioral physiological parameters through noninvasive or minimally invasive interfaces with the human body, typically implemented on body-conformal platforms. These sensors merge biomedical sensing, data processing, and wireless communication, representing a transformative shift from traditional point-of-care diagnostics toward continuous digital health surveillance and tracking [1,2,3]. Advances in semiconductor fabrication, electronics miniaturization, and 3D printing technologies have enabled the development of compact, battery-powered wearable devices for consumer applications. The emergence of first-generation fitness trackers in the early 2000s, which are primarily based on accelerometers and optical sensors, have expanded applications beyond clinical cardiology to general wellness, activity tracking, and sleep monitoring [4,5]. These early devices, however, were largely limited to biophysical sensing. Recent breakthroughs in flexible materials, bio-integrated electronics, and soft mechanics have driven the evolution toward “second-generation” wearables that achieve seamless skin conformity, improved biocompatibility, and multimodal sensing of both physical and biochemical parameters [1,6,7,8]. Skin-interfaced systems and epidermal electronics now enable continuous, high-fidelity measurement of electrophysiological, biochemical, and mechanical signals while maintaining user comfort [2,6]. These sensing platforms are often integrated with nanomaterials, conductive polymers, and intrinsically stretchable substrates, which facilitate real-time signal capture and wireless data transmission [3,7].

The integration of wearable biosensors with artificial intelligence (AI) and machine learning (ML) represents a shift from passive data collection to computational interpretation of continuous physiological and biochemical signals. In this setting, AI/ML models can detect patterns in electrocardiography (ECG), photoplethysmography (PPG), inertial measurement unit (IMU), temperature, and sweat-biomarker streams and translate them into clinically relevant outputs such as arrhythmia alerts, activity and gait metrics, stress-state estimates, exposure risk, or biomarker trends. AI-enabled systems can identify disease-specific patterns, enhance predictive accuracy, and support personalized healthcare strategies [1,2]. The trajectory from basic wearable fitness and vital-sign monitors to AI-integrated bioelectronic platforms thus reflects the ongoing shift from episodic medical testing to continuous, intelligent health monitoring and diagnosis [1,2]. Despite the rapid growth in AI-enabled wearable sensing, existing reviews often remain organized around individual sensing modalities or application areas, with limited cross-domain comparison of how models are evaluated and translated toward deployment. In particular, reported performance is frequently summarized using headline metrics without sufficient discussion of dataset scale, subject-level validation, controlled versus real-world settings, computational constraints, interpretability, or regulatory readiness. This creates a need for a review that not only summarizes representative applications, but also evaluates the methodological rigor and translational maturity of wearable AI systems.

To address this gap, this review provides a cross-domain synthesis of AI-integrated wearable sensing systems. Specifically, we (i) summarize major physiological, behavioral, environmental, and biochemical sensing modalities; (ii) compare representative AI/ML studies across application domains using standardized tables that report task formulation, model type, dataset characteristics, validation protocol, and performance metrics; (iii) discuss advanced architectures, multimodal fusion, hardware–software co-design, and explainable AI for wearable health applications; and (iv) identify key barriers to clinical and commercial translation, including privacy, regulatory requirements, sensor drift, calibration burden, cross-subject generalization, and post-deployment monitoring.

## 2. Physiological and Biochemical Sensing Modalities in Wearable Sensors

Wearable sensors have three sensing paradigms: motion and activity tracking, vital-sign monitoring, and biochemical sensing [1]. Motion and activity trackers are typically built on inertial measurement units (IMUs) that integrate accelerometers and gyroscopes (often with magnetometers) to quantify gait, posture, and daily activities in real-world settings and rehabilitation contexts [9]. System-level frameworks situate these motion sensors within end-to-end wearable architectures, including substrates, sensing/transduction modules, microcontroller units, and power modules, enabling interoperable designs across clinical and consumer environments [1].

Vital-sign monitors comprise optical and electrical sensors for cardiovascular and cardiorespiratory assessment, with photoplethysmography (PPG) widely used to derive heart rate, heart-rate variability (HRV), and sleep-related metrics while contending with motion artifacts and skin-tone/physiology confounders [10]. Ambulatory cardiac monitoring now spans adhesive electrocardiography (ECG) patches and smartphone-linked wearables for rhythm surveillance, expanding access to remote diagnostics and longitudinal arrhythmia assessment outside the clinic [11]. Cuffless blood pressure tracking also illustrates potential for routine clinical adoption [12].

Biochemical sensors extend wearables beyond biophysical parameters by sampling accessible biofluids such as sweat, where flexible electrochemical platforms, often coupled with soft microfluidics, enable continuous, multiplexed analysis of electrolytes and metabolites for personalized monitoring and stress/exertion assessment [8]. This biochemical layer is increasingly integrated into standardized data frameworks and decision-making units to support real-time detection and intervention [1]. For example, continuous glucose monitoring (CGM) provides standardized clinical endpoints and validation metrics that support trials, practice guidelines, and closed-loop diabetes management [13].

With their advanced sensing capabilities, wearable sensors play a critical role in remote patient monitoring (RPM) and telemedicine by enabling continuous clinical observation outside traditional healthcare environments and supporting safe transitions from hospital to home care [14]. RPM interventions leveraging connected sensors, smartphone gateways, and clinician dashboards generally improve patient safety and adherence, with consistent signals for reduced admissions, length of stay, outpatient visits, and costs during care transitions [14]. Health-system and nursing perspectives similarly underscore telehealth + RPM as a maturing standard of care that creates data-rich, bidirectional patient–provider workflows when paired with appropriate governance and best practices [15]. Additionally, personalized treatment paradigms increasingly depend on continuous, high-resolution physiological data from wearables to tailor clinical decisions to each patient’s condition [15]. Wearable sensors also play an important role in chronic disease management by enabling continuous monitoring that supports proactive therapy adjustment, self-management, and earlier detection of clinical deterioration. For example, in heart failure (HF), wearable sensing technologies can help reduce hospital readmission risk and improve quality of life when technical and implementation challenges are addressed [16]. Wearable sensors can also contribute to early infectious disease detection: deviations from individual physiological baselines—such as resting heart rate, sleep patterns, and activity levels—have been shown to signal COVID-19 infection prior to symptom onset, enabling earlier testing and isolation [17,18]. Collectively, these advances position wearable sensors as key components of modern healthcare systems, supporting remote patient monitoring and telemedicine while enabling pre-symptomatic detection, personalized therapy, and improved management of chronic diseases through continuous and interpretable data streams [5,13,14,15,16,17,18,19,20].

## 3. Enhancing Wearable Sensors with Artificial Intelligence (AI)

Artificial intelligence (AI) is a system-level approach that covers perception, reasoning, decision-making, and interaction, and it can combine learned models with heuristic procedures [21]. Within this discipline, machine learning (ML) is the subfield that enables systems to improve task performance from data rather than explicit programming, and deep learning (DL) is the subfield of ML that uses multi-layer neural networks to learn representations from large datasets. Given these distinctions, traditional machine learning-based models are well-suited when datasets are modest, features are low-dimensional or hand-crafted, and interpretability or calibrated probabilities are required [22,23]. When problems involve large datasets or high-dimensional signals such as images, raw time series, audio, or text, or when feature interactions are complex and hierarchical, practitioners typically employ deep learning-based approaches that learn representations end to end through convolutional, recurrent, or transformer architectures [24]. Deep learning often yields higher accuracy under these conditions but generally requires more data and compute and provides less transparency, so explanation methods or hybrid pipelines are used when interpretability is needed [25].

Wearable sensing represents a key application domain for artificial intelligence [26]. In wearable sensing, AI techniques process raw physiological signals acquired by wearable sensors to derive clinically useful information for inferring events, states, and risk estimates. In practice, they translate electrocardiography (ECG), photoplethysmography (PPG), inertial measurement unit (IMU) signals, respiration, temperature, and biochemical streams into outcomes such as arrhythmia detection, activity and gait metrics, stress episodes, and exposure dose. Because these techniques unify perception, learning, and decision making over multimodal, high-frequency data, they have significantly advanced personalized clinical decision support systems (CDSSs) and enabled sensor-based diagnosis and measurement, as discussed in [27,28].

Operationally, AI pipelines proceed through data acquisition, preprocessing, feature engineering, model inference, and optional post-processing and integration. Because machine learning-based models are data-driven, their performance depends critically on dataset quality. Wearable signals often contain motion artifacts, sensor noise, drift, and missing segments; as detailed in [28,29], effective preprocessing includes signal filtering, noise reduction, artifact removal, data normalization and scaling, and imputation of missing values. Feature engineering transforms raw measurements into variables that models can use effectively [30]. It comprises feature extraction, which derives descriptors from the data, and feature selection, which chooses a compact subset with high predictive value. In wearable sensing, common extracted descriptors include ECG and PPG heart rate and interbeateat intervals, heart rate variability (HRV) statistics, frequency band powers and spectral entropy, morphology-based metrics, IMU statistics and gait markers, respiratory rate and variability, and temperature or biochemical trends [31]. Selection is typically performed with filter criteria, wrapper searches, or embedded methods, evaluated within nested cross validation, while deep learning can learn representations end to end and therefore reduces the need for handcrafted features on large datasets, preprocessing remains essential, and engineered features are still valuable for small data regimes and for interpretability [30]. Post-processing can smooth predictions, enforce physiological constraints, and route results to CDSSs [32,33].

**Figure 1:**
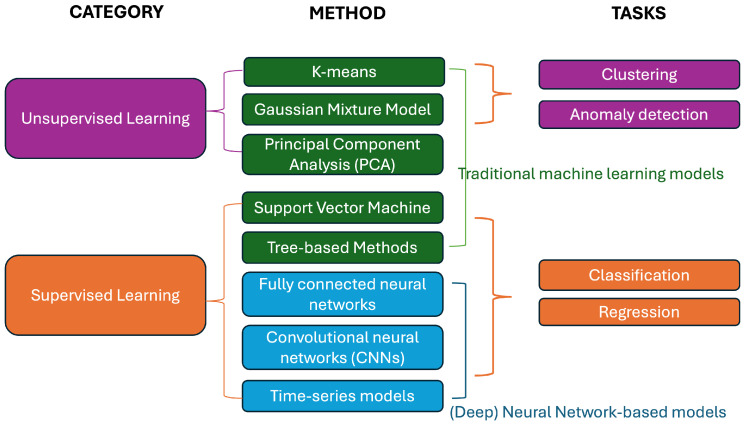
Machine learning paradigms and representative models. Different colors distinguish learning categories, representative methods, and associated task types.

Methodologically, the machine learning paradigms and representative models used in this domain are summarized in Figure 1. Supervised learning trains on labeled examples to learn functions from inputs to targets and supports tasks such as classification and regression [34]. Unsupervised learning operates without labels to uncover structure in the inputs, with common tasks including clustering, dimensionality reduction, density modeling, and anomaly detection [35]. In practice, unsupervised objectives are often used during pre-training to learn representations and to assess signal quality. Traditional machine learning models [23,36] (e.g., linear and logistic regression, support vector machines (SVMs), and tree-based ensembles such as random forests and gradient boosted trees) are comparatively transparent: coefficients, margins, and split rules expose feature importance, and many models can be calibrated to yield probability estimates. By contrast, neural network-based models (e.g., fully connected networks, convolutional networks, and time series architectures) often achieve higher accuracy on large datasets but offer less transparency; post hoc explanation methods, such as feature attribution and local surrogate models, provide local interpretability when clinical review is required [31,32,37].

In practice, model selection depends on data volume, label quality, privacy constraints, and deployment limits. When datasets are small or privacy prevents broad data sharing, simpler models such as linear or logistic regression and tree-based ensembles tend to be more stable and easier to calibrate and explain [38,39]. When large and diverse labeled datasets are available and the task requires complex pattern recognition, deep neural networks usually provide better accuracy. With limited data, the risks of overfitting and underfitting increase, so the proper use of regularization, cross-validation, early stopping, and careful calibration becomes especially important [40,41].

**Figure 2:**
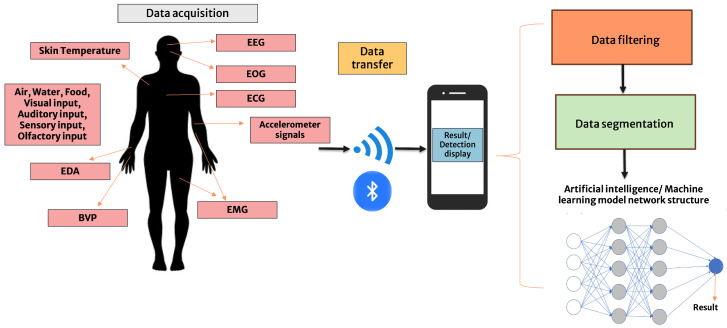
Diverse physiological and biochemical signals collected by wearable sensors from the human body serve as input data for AI/ML models to enhance diagnostic accuracy in healthcare.

Wearable sensing has become tightly coupled with AI, and current research extends beyond single modality analysis (Figure 2). Current advances explore multi-modal fusion of heterogeneous signals, privacy-preserving learning frameworks such as federated learning, and efficient on-device inference to meet the constraints of mobile and embedded systems [42,43]. Reliable use requires precise task definition, prevention of data leakage, and rigorous validation with appropriate metrics, external testing, and fairness assessment [44]. By mitigating overfitting and ensuring transparency, these practices enable trustworthy and clinically valuable deployment of AI-enabled wearable sensing [45].

### 3.1. Advanced Architectures for Temporal and Multimodal Wearable Data

While convolutional neural networks (CNNs) and recurrent neural networks (RNNs) have dominated wearable signal modeling, two architectural classes have recently attracted increasing attention: Transformer networks and generative adversarial networks (GANs). CNNs are effective for extracting local morphological features but may require deep, dilated, or multi-scale designs to capture long-range temporal dependencies, while RNNs process sequences serially, which can limit training parallelism and make very long-range dependencies difficult to preserve [46]. Wearable sensing also faces persistent data limitations, including small labeled cohorts, class imbalance for rare pathological events, and privacy constraints on data sharing, which can restrict the deployment of deep learning at clinical scale [47,48]. Transformers and GANs offer complementary solutions to these challenges: transformers improve representation learning under long-range and multimodal dependencies, whereas GANs help mitigate data scarcity, imbalance, and privacy constraints through synthetic data generation.

Transformer networks, originally introduced by Vaswani et al. for sequence modeling in natural language processing [49], replace recurrence with a self-attention mechanism in which each token in a sequence is compared with other tokens in parallel, producing a global, content-aware weighting of dependencies. For wearable signals, this property is particularly useful because attention can directly relate samples separated by many timesteps, which is challenging for conventional convolutional or recurrent models. Empirically, transformer-based models have reported improvements over CNN–LSTM baselines on accelerometer- and gyroscope-based human activity recognition benchmarks [50], and have achieved comparable or improved performance relative to ResNet- and long short-term memory (LSTM)-based architectures on single-lead electrocardiogram (ECG) arrhythmia classification by jointly modeling intra-beat morphology and inter-beat rhythm structure [51]. Self-attention also provides a flexible mechanism for multimodal fusion, allowing models to learn token-level interactions across heterogeneous streams such as ECG, photoplethysmography (PPG), respiration, and inertial signals without relying entirely on hand-designed fusion rules. The principal drawback of canonical transformers is the quadratic computational and memory complexity of self-attention with respect to sequence length, which can limit direct deployment on resource-constrained wearable hardware [46]. Recent studies have addressed this limitation through architectural compaction, efficient attention variants, and recurrent–transformer hybrids. For example, the Lightweight Fussing Transformer replaces standard self-attention with a LightConv Attention mechanism and reduces parameter count by approximately 72% relative to the baseline transformer while preserving heartbeat classification performance on dynamic ECG [52]. Similarly, lightweight sequential transformers that combine attention with recurrent processing have been proposed for blood glucose prediction on edge devices [53]. These efficiency-oriented designs reflect the broader trend toward making transformer-based wearable models more practical for ambulatory and edge-device settings.

Generative adversarial networks, introduced by Goodfellow et al. [54], consist of a generator that synthesizes data and a discriminator that distinguishes real from synthetic samples, with the two networks trained through an adversarial objective. In wearable sensing, GANs have been applied primarily to data augmentation and privacy-preserving synthetic data generation. Class imbalance is common in clinical wearable datasets: rare arrhythmia classes, stress episodes, and adverse glycemic events are often substantially underrepresented relative to majority classes, biasing classifiers toward dominant categories and reducing sensitivity to clinically important events [47]. Conditional GANs address this issue by conditioning the generator on class labels, enabling the synthesis of labeled minority-class samples for training augmentation. A systematic review of GAN-based ECG synthesis reported classification accuracy improvements of approximately 6–7 percentage points when GAN-generated signals were added to imbalanced training sets across thirty studies [47]. Similar benefits have been reported for stress recognition from multimodal physiological data, where a conditional GAN combining a long short-term memory generator with a fully convolutional discriminator and an explicit diversity term produced synthetic streams that improved downstream classifier performance and showed close agreement with real data under expert evaluation [55]. A further advantage for medical wearables is privacy-preserving synthesis: differentially private GANs (DP-GANs) can generate synthetic signals that preserve dataset-level statistical properties while reducing exposure of individual records. In a wearable stress detection task, this approach improved F1-score by 11.90 to 15.48 percentage points under differential privacy constraints relative to training on the limited original data alone [48]. Despite these benefits, GAN-based approaches remain challenging because of training instability, mode collapse, and the lack of standardized quality evaluation metrics for synthetic biosignals, which complicates fair comparison across studies [47].

Overall, transformers and GANs address complementary challenges in wearable AI: transformers improve representation learning for long-range and multimodal temporal dependencies, while GANs help mitigate data scarcity, imbalance, and privacy constraints through synthetic data generation. Emerging hybrid directions, including transformer-based generators or attention-based discriminators, suggest that these architectures may be combined to synthesize physiologically plausible signals while preserving long-range temporal structure. Large-scale pretraining on multimodal or unlabeled wearable corpora is also emerging as a related direction for improving transfer across downstream sensing tasks. Nevertheless, both architectures still face limitations in computational cost, interpretability, and standardized evaluation under realistic deployment conditions. Their practical impact in wearable health monitoring will therefore depend not only on architectural innovation, but also on transparent reporting, deployment-aware validation, and integration with preprocessing, calibration, and post-processing pipelines.

Beyond architectural compaction, hardware–software co-design is increasingly important for deploying AI on resource-constrained wearable platforms. Model-level techniques such as weight pruning, low-precision quantization, and knowledge distillation can reduce memory and computational demands while preserving task performance [56,57,58]. Hardware-level approaches, including TinyML frameworks, low-power microcontrollers, and specialized accelerators, support on-device inference under strict energy, memory, and latency constraints [59,60]. Edge–cloud hybrid inference provides a complementary strategy, in which lightweight on-device models perform continuous screening and trigger cloud-based analysis only when higher-capacity inference is needed, balancing latency, accuracy, privacy, and energy consumption [61,62].

### 3.2. Multimodal Fusion Strategies for Wearable Health AI

Wearable health AI systems increasingly combine heterogeneous data streams—physiological signals (ECG, PPG, HRV, EDA), motion data (accelerometer, gyroscope), environmental context (temperature, humidity, ambient pollutants), and contextual variables (time of day, activity, self-report)—to improve diagnostic accuracy and predictive performance [63]. Fusion strategies for these multimodal streams fall into three broad categories distinguished by where the fusion occurs in the inference pipeline [64]. Early fusion (data-level) concatenates raw or lightly preprocessed signals before model input and is simple but sensitive to differences in sampling rate, scale, and missing modalities. Mid-level fusion(feature-level) extracts modality-specific representations using parallel encoders and combines them at an intermediate layer, providing greater flexibility in handling heterogeneous data types. Late fusion (decision-level) trains separate models on each modality and combines their outputs through voting, weighted averaging, or stacking, which improves robustness to missing modalities but loses cross-modal interactions. Attention-based fusion, enabled by Transformer architectures, has emerged as a fourth strategy that learns dynamic, sample-specific weights for each modality and naturally handles missing or low-quality streams.

Representative examples from the reviewed literature illustrate the practical impact of fusion strategy: HRV and EDA combined with skin temperature for stress detection benefits from mid-level fusion that preserves the autonomic-specific signatures of each signal [65]; ECG combined with PPG and accelerometer for ambulatory cardiac monitoring uses attention-based fusion to weight different modalities according to motion-artifact severity; and physiological signals combined with self-reported symptoms for infectious disease surveillance benefits from late fusion to accommodate the discrete and intermittent nature of symptom reports [66]. Effective multimodal fusion in wearable AI also requires careful handling of three practical issues—temporal alignment across modalities with different sampling rates, missing-modality robustness when individual sensors fail or are not worn, and uncertainty quantification across modalities of differing reliability—which together determine whether multimodal benefits translate from controlled studies to deployment.

Across these architectures, model interpretability is an important design consideration for clinical deployment. The choice of explainable AI (XAI) method should depend on the model architecture, the required explanation granularity, and the intended clinical audience. SHAP (SHapley Additive exPlanations) [67] provides additive feature attributions and is well suited to tree-based ensembles and tabular wearable features; in wearable applications, it has been used to identify HRV and EDA as important predictors of stress state and to highlight time-from-midnight as a circadian feature in wearable glucose prediction [68]. LIME (Local Interpretable Model-agnostic Explanations) [69] trains a local surrogate model around an individual prediction and is useful when sample-specific, model-agnostic explanations are needed. For deep models operating on raw biosignals, Integrated Gradients [70] can attribute predictions to input features by integrating gradients from a baseline, while Grad-CAM [71] highlights input regions that contribute strongly to CNN predictions, making it useful for ECG and image-based wearable tasks. For attention-based architectures such as Transformers, attention weights can provide additional cues about influential tokens or modalities, although they should be interpreted cautiously as explanatory evidence. In practice, hybrid explanation strategies, such as combining SHAP-based global feature importance with waveform- or image-level heatmaps, can better support clinical review by linking overall feature trends to local evidence in individual predictions.

## 4. AI-Enabled Physical Activity Recognition

**Figure 3:**
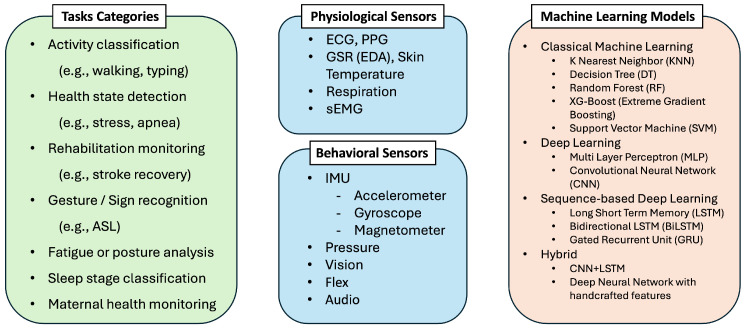
Overview of task categories, sensor modalities, and machine learning models in wearable activity recognition.

Physical activity recognition (PAR) using wearable sensors has emerged as a foundational capability for health monitoring, rehabilitation, occupational safety, and wellness management [72,73]. The sensing targets span a broad spectrum, from coarse daily activities such as walking, sitting, and climbing stairs, to fine-grained movements such as gestures and postural transitions [74,75,76]. Correspondingly, the downstream AI tasks are predominantly formulated as multi-class classification problems, though regression tasks such as step-length or gait-parameter estimation are also explored for clinical assessment [77]. The clinical and operational relevance of accurate PAR is significant: continuous rehabilitation monitoring can ensure patient compliance and accelerate recovery [78,79], early detection of hazardous postures can reduce occupational injury risk [80,81], and sleep or hydration classification can support proactive chronic disease management [82,83]. Figure 3 presents an overview of the task categories, sensor modalities, and machine learning models commonly employed in this domain.

### 4.1. Human Activity Recognition

The core challenge in human activity recognition (HAR) is learning robust activity representations from wearable sensor streams that vary considerably across individuals, environments, and device placements. Input modalities range from inertial measurement units (IMUs) and strain sensors to biometric and environmental signals, and the choice of modality critically determines what activities can be reliably discriminated.

For gesture and fine-grained motion recognition, strain-based sensors that capture 3D resistance signals from key joints have been combined with multilayer perceptron (MLP) classifiers refined via fuzzy inference, achieving high regression fidelity ($R^{2}=0.9286$) for continuous strain estimation under a weakly supervised multi-class formulation [84]. For broader daily activity recognition, inertial sensing combined with sliding-window statistical feature extraction and random forest classification has demonstrated over 94% accuracy across 13 indoor and outdoor activities, including typing and folding laundry, under 10-fold cross-validation [85]. When environmental context is incorporated alongside biometric signals such as heart rate and step count, annotation granularity becomes a critical factor: minute-level self-reporting significantly outperforms coarse hourly logs, with random forest and decision trees reaching up to 77% accuracy in an urban participatory exposure study with 18 participants [86]. For maternal activity classification using triaxial accelerometer, gyroscope, and temperature signals from a wrist-worn module on pregnant participants, gradient boosted trees achieved the highest accuracy at 89% under a 90/10 train-test split, though random forest was preferred for deployment due to its lower computational overhead [87].

### 4.2. Sleep Monitoring

Sleep monitoring from wearable sensors spans multiple task formulations depending on the clinical objective, ranging from binary classification of disordered breathing and multi-class sleep-stage estimation aligned with American Academy of Sleep Medicine (AASM) guidelines, to regression-based sleep-quality scoring and anomaly detection. The input typically comprises heart rate, oxygen saturation, respiration rate, and accelerometer signals from consumer smartwatch platforms. A persistent challenge across all formulations is that class imbalance, limited temporal context, and fine-grained boundaries between adjacent sleep stages collectively degrade multi-class performance, making model architecture and evaluation protocol particularly consequential.

For sleep apnea detection formulated as binary classification from heart rate and triaxial accelerometer signals on real patients, an MLP with three hidden layers trained on smartwatch-compatible signals achieved 92% accuracy, while Gaussian Naive Bayes showed competitive performance at 89%, with multi-class severity estimation degrading substantially to 71%—a drop directly attributable to class imbalance and the absence of sequential modeling [83]. For three-class detection of workout, awake, and sleep states, multiparameter fusion of wearable features using SVM and random forest both achieved F1-scores above 0.96 on longitudinal data (approximately 64 days, 57 workout sessions) collected from a single participant [88]. F1-score is the preferred evaluation metric in imbalanced sleep datasets, while accuracy is reported where class distributions are approximately balanced.

### 4.3. Posture Classification

Posture and gesture recognition share the challenge of modeling fine-grained, high-dimensional body movement from wearable sensor streams, but differ substantially in the temporal structure of the input and the granularity of the target label.

Gesture recognition from hand-mounted sensor fusion, combining flex sensors, pressure sensors, and IMU data processed through a sliding window SVM classifier, has achieved over 95% accuracy across 27 American Sign Language classes trained on over 6.4 million labeled instances from 12 subjects under leave-one-out cross-validation [76]. For posture classification in occupational settings, time-series plantar pressure data from wearable insoles segmented with overlapping windows have been used to predict musculoskeletal strain-related posture labels during rebar tasks collected on an active construction site, with gated recurrent unit (GRU) achieving 99.01% accuracy and outperforming both LSTM and bidirectional LSTM variants [81]. Together, these results suggest that sensor fusion is critical for gesture recognition, while temporal modeling with recurrent networks is better suited for posture classification in physically demanding environments.

### 4.4. Rehabilitation Monitoring

Rehabilitation monitoring and early disease detection represent high-stakes applications where wearable AI systems must generalize across individuals with highly variable motor patterns and physiological responses.

For stroke rehabilitation, personalized CNN models trained on commercial smartwatch IMU signals from 17 chronic stroke survivors achieved 99.9% accuracy under five-fold cross-validation on within-subject personal data, substantially outperforming the cross-subject (total-data) model that reached 95.8% on the same task, highlighting the importance of individual-specific calibration for recovery support [78]. For shoulder rehabilitation, a random forest trained on statistical features extracted from three magneto-inertial sensors worn by 19 healthy participants and 17 patients with rotator cuff tears achieved perfect classification across six targeted exercises under 5-fold cross-validation [79]. Beyond rehabilitation, CatBoost (a gradient boosting variant) applied to capacitance signals from a flexible multi-sensor bra system achieved 95% accuracy for breast cancer screening, with single-sensor configurations performing substantially worse, underscoring the impact of sensor density on detection reliability [89]. Multispectral sensor fusion incorporating photoplethysmography signals at 970 and 1450 nm with smartwatch IMU data, processed through LightGBM under four-fold cross-validation on data from 19 participants performing 103 indoor 5-km treadmill trials under controlled ambient conditions (10–34 °C, 25–60% relative humidity), demonstrated the feasibility of continuous hydration monitoring via binary classification of pre- and post-exercise states [90].

### 4.5. Motor Disorder Monitoring

Motor disorder monitoring using wearable IMU and EMG signals is often formulated as a classification problem, in which the target corresponds to a clinically defined movement abnormality, such as postural instability or tremor. However, depending on the clinical objective, the problem can also be formulated as regression to estimate symptom severity, event detection to identify abnormal movement episodes, or time-series prediction to forecast motor impairments. The input is typically a multimodal time-series stream, and the challenge lies in distinguishing pathological movement patterns from normal variability with high sensitivity and specificity, as misclassification carries direct clinical consequences.

For Parkinson’s disease (PD) postural instability detection using body-mounted IMUs, fine-tuned k-nearest neighbors achieved 95.6% accuracy when distinguishing healthy controls from PD patients, with near-perfect recall; performance dropped to 84.2% accuracy when classifying ON versus OFF medication states within the same cohort, illustrating that classifier accuracy depends heavily on which contrast is evaluated [91]. For tremor classification using EMG and accelerometer fusion from wrist and hand sensors, bagged decision trees achieved up to 99.6% accuracy as the best result among six compared classifiers (decision tree, k-NN, linear discriminant analysis (LDA), SVM, boosted trees, and bagged trees), demonstrating the effectiveness of multimodal ensemble learning for fine-grained motion analysis in clinical populations [92]. For real-time gait analysis using a single lower-back IMU, XGBoost achieved the lowest RMSE for step length estimation (below 5 cm averaged over ten consecutive steps, intraclass correlation coefficient (ICC) (2,1) = 0.93) across a diverse cohort of 472 subjects spanning Parkinson’s disease, mild cognitive impairment, multiple sclerosis, healthy young adults, and older adults, with more than 80,000 individual steps analyzed—confirming that compact sensor setups can yield clinically relevant gait metrics with appropriate regression modeling [77].

### 4.6. Evaluation Methodology and Generalizability in PAR

Across the PAR studies summarized in Table 1, three evaluation patterns recur. First, within-cohort k-fold cross-validation is widely used because it provides a convenient estimate of model performance on the available dataset [79,85,90]; however, when samples from the same subject appear in both training and test folds, the resulting accuracy may be optimistic for deployment on unseen users. Second, within-subject personalized evaluation is appropriate for rehabilitation and patient-specific monitoring [78,88], but these results should be interpreted separately from cross-subject performance. Third, subject-independent protocols, such as leave-one-subject-out evaluation or testing across heterogeneous cohorts [76,77], provide stronger evidence of generalizability. Future PAR studies should therefore report the data-partitioning protocol explicitly, distinguish subject-level from record-level splits, and include cross-device or cross-subject evaluation when feasible.

Across PAR domains, the reviewed studies show that reported performance is shaped by activity-set complexity, cohort size, sensor configuration, class balance, and validation protocol. While shallow ensemble methods remain competitive for structured, low-dimensional inputs, recurrent and hybrid deep architectures are better suited for capturing the temporal complexity of sequential physiological and motion data [93,94]. Future progress will require balanced data collection, transparent evaluation protocols, and model designs that remain robust across individuals, devices, and real-world deployment contexts.

## 5. AI-Driven Stress and Anxiety Monitoring

Stress and anxiety manifest through measurable physiological and behavioral changes, including alterations in heart rate variability, electrodermal activity (EDA), skin temperature, and motor patterns, that can be continuously captured by wearable sensors. Unlike discrete clinical events such as arrhythmia or epileptic seizures, stress is a graded, subjective state influenced by individual physiology, context, and population characteristics, making robust label acquisition and model generalization particularly challenging. The integration of AI with multimodal wearable sensing offers a pathway toward real-time, personalized stress monitoring across everyday and clinical contexts.

### 5.1. Stress-Level Classification

Stress classification from wearable sensors is predominantly formulated as a binary or multi-class problem, where the input is a multimodal physiological or behavioral time-series stream and the output is a discrete stress-level label. A core challenge is the high inter-individual variability of stress responses: identical physiological changes (for example, a comparable rise in heart rate or skin conductance) can reflect different stress levels across individuals depending on baseline autonomic tone, fitness, comorbidities, and contextual factors. As a consequence, no single signal modality has been shown to provide universally reliable discrimination across populations and contexts, motivating the multimodal sensing strategies described in the remainder of this section [65,95,96].

Physiological signals, particularly HRV analyzed in the time- and frequency-domains and nonlinear indices, have been established as among the most informative indicators of autonomic nervous system response to stress, with skin temperature providing complementary evidence of sympathetic activation [95]. Building on this, multimodal fusion has been shown to improve classification robustness in occupational settings. In particular, Antwi-Afari et al. [96] combined plantar pressure and acceleration signals from an insole-based wearable with a structured feature engineering pipeline spanning frequency-domain, time-domain, and spatiotemporal descriptors. Their random forest classifier achieved 86% overall accuracy for multi-class physical fatigue and stress classification in construction workers under 10-fold cross-validation; per-class metrics span a wider range than the aggregate, with precision 52.6–82.6%, recall 52.6–84.3%, specificity 89.6–92.3%, and F1-score 52.6–83.5% across the three physical-fatigue-level sub-problems [96], consistent with class imbalance and macro-averaging in this multi-class setting.

For behavioral sensing, keystroke dynamics captured via a smartphone accelerometer and a gyroscope have also shown discriminative potential for binary stress classification, with k-NN achieving 87.56% accuracy on data from 46 participants performing typing tasks across both stress and calm conditions [97]. Integrating facial emotion recognition with ECG-based physiological stress detection addresses the ambiguity of visual-only systems, reinforcing emotional inferences with autonomic cues and achieving 83.33% accuracy for negative emotion and stress-state detection [98].

For higher-accuracy inference, stacked ensemble architectures that aggregate predictions from random forest and gradient boosting base models via a meta-learner have reported 99.5% accuracy on body temperature, humidity (sweat), and step count inputs for three-class (low/normal/high) stress classification [99]; this figure was obtained on the publicly available Stress–Lysis benchmark (2001 samples) using a simple 20% train–test holdout, and the original study did not collect new wearable measurements from real participants, so the number should be interpreted as a benchmark upper bound rather than evidence of real-world deployment performance. For sequential biosignals such as EDA and blood volume pulse (BVP)-derived HRV, three-layer stacked LSTM networks with dropout regularization capture temporal dependencies that shallow models cannot, achieving area under the curve (AUC) 0.90 and outperforming logistic regression by approximately 11% for stress classification in 19 older adults under a Trier Social Stress Test (TSST) protocol with salivary cortisol as the physiological ground truth [65]. Together, these results suggest a clear architecture–task alignment: ensemble methods are well-suited to structured, multimodal inputs with engineered features, while recurrent deep models are better-suited to raw sequential physiological streams where temporal dynamics carry discriminative information.

### 5.2. Mental Health Assessment

Mental health assessment in clinical populations introduces additional constraints beyond general stress classification: labels are often derived from subjective or sparse clinical annotations, physiological responses are more variable due to comorbidities and medication effects, and model interpretability is critical for clinical acceptance. The task is typically formulated as binary or multi-class stress state classification from physiological signals, including HR, HRV, and EDA, collected during structured therapeutic sessions. In older adults with mild cognitive impairment (MCI), random forest and AdaBoost trained on HRV and EDA features selected via correlation-based feature selection, information gain ratio, and PCA achieved accuracies of 85.4% and 85.3% respectively, with area under the precision-recall curve (AUPRC) values of 0.98 and 0.97, demonstrating that interpretable ensemble methods can deliver reliable and clinically transparent stress monitoring in vulnerable populations [100]. AUPRC is particularly appropriate here given the class imbalance inherent in clinical stress datasets, where stress episodes are less frequent than baseline states.

### 5.3. Evaluation Methodology and Generalizability in Stress Monitoring

Evaluation in stress monitoring depends strongly on label quality, dataset provenance, and validation protocol, as summarized in Table 2. First, subjective self-reports are useful but noisy, whereas physiological references such as salivary cortisol under a Trier Social Stress Test protocol provide a more objective basis for model evaluation [65]. Second, results obtained on public benchmark datasets or small single-source datasets should be interpreted as benchmark performance unless external validation or newly collected participant data are reported [99]. Third, because stress labels are often imbalanced and population-dependent, AUC, AUPRC, and per-class precision/recall/F1 are generally more informative than raw accuracy alone. Future stress-monitoring studies should clearly describe label acquisition, report class-wise metrics, and evaluate cross-subject or cross-population performance where feasible.

Across the reviewed studies, stress-monitoring systems rely on diverse wearable platforms, ranging from smartphones and insoles to body-worn sensors, capturing physiological signals such as HRV and EDA alongside behavioral signals such as keystroke dynamics and plantar pressure. Shallow ensemble methods perform well on structured, feature-engineered inputs, while LSTM-based architectures are better suited to sequential biosignals where temporal dynamics carry discriminative information [65,96]. Future work should focus on standardized labeling protocols, clinically interpretable model designs, and validation across broader populations.

## 6. AI-Driven Continuous Cardiovascular Monitoring

**Figure 4:**
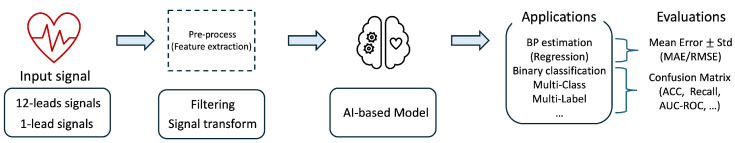
Overview of ECG-based AI applications using wearable devices.

Continuous and accurate monitoring of vital physiological signals, particularly electrocardiographic (ECG) activity and blood pressure (BP), is essential across a broad range of clinical applications. These signals serve as key indicators not only of cardiovascular health, but also of neurological, renal, and metabolic conditions, making their reliable acquisition and interpretation critical for early diagnosis, disease management, and patient safety [102,103,104]. ECG measures the heart’s electrical activity by detecting small potential changes on the body surface caused by the depolarization and repolarization of cardiac muscle during each cardiac cycle [102], while BP reflects the hemodynamic load on arterial walls, providing complementary insight into circulatory function. Despite their clinical importance, conventional rule-based ECG interpretation systems exhibit significant error rates in complex diagnostic scenarios [105], and traditional BP measurement remains cuffed, intermittent, and impractical for continuous use. The integration of AI with wearable sensor technologies offers a promising pathway to overcome these limitations, enabling real-time, unobtrusive, and personalized physiological monitoring [106]. Figure 4 illustrates a generalized pipeline for such systems, from signal acquisition through AI model processing to task-specific outputs such as arrhythmia detection and BP estimation, evaluated using metrics including RMSE, AUC, and F1-score.

### 6.1. ECG Monitoring

The primary challenge in wearable ECG monitoring lies in accurately interpreting complex cardiac rhythms from signals that are inherently noisy and limited in lead configuration. Unlike clinical 12-lead systems, wearable devices typically rely on single-lead recordings [107], which capture cardiac electrical activity along a single spatial axis, offering less diagnostic coverage than multi-lead clinical systems and are more susceptible to motion artifacts and baseline wander. Although advances in textile-based electrode design have improved acquisition comfort by embedding electrodes directly into garments [108], the fundamental signal quality constraints remain. Furthermore, conventional rule-based interpretation systems have shown significant limitations in handling the diversity of arrhythmia morphologies [105], motivating the increasing adoption of AI and machine learning approaches that can learn discriminative features directly from raw signals [106].

The core task is formulated as a multi-class classification problem, where the input is a raw or minimally preprocessed single-channel ECG time series and the output is a predicted rhythm label. A key modeling challenge is that ECG interpretation requires the simultaneous understanding of local waveform morphology—such as P-, Q-, R-, S-, and T-wave shapes—and long-range temporal dynamics across cardiac cycles. To address this, CNN-based architectures have been widely adopted for local feature extraction, often combined with RNN or LSTM layers to capture sequential dependencies in CNN–RNN hybrid designs [109]. Pure deep-CNN architectures without explicit recurrent components have also proven effective; for example, Hannun, Rajpurkar et al. trained a 34-layer convolutional neural network on 91,232 single-lead ECG records from 53,549 patients collected with the Zio Patch ambulatory monitor, classifying 12 rhythm classes with an average AUC of approximately 0.97 against a cardiologist consensus reference [110]. These approaches have proven effective across tasks of varying complexity, from binary arrhythmia detection to multi-class rhythm classification with up to 20 classes [109], consistently achieving receiver operating characteristic (ROC)-AUC scores above 0.90 on real-world wearable data [109,110].

AUC and ROC-AUC are the dominant evaluation metrics in this domain, largely because arrhythmia datasets are heavily class-imbalanced, making accuracy an unreliable performance indicator.

### 6.2. Blood Pressure Monitoring

Continuous BP estimation from wearable sensors is fundamentally formulated as a regression problem, where the input is a physiological waveform signal and the output is a continuous scalar value representing systolic BP (SBP) or diastolic BP (DBP) in mmHg. The primary challenges are twofold: the relationship between surface physiological signals and arterial pressure is indirect and nonlinear, and substantial inter-individual variability in vascular properties means that a generalized model may perform poorly on unseen individuals without personalization.

Two principal input modalities have been explored to address these challenges. The first uses single-channel ECG signals, which encode pulse-transit information that is indirectly related to arterial pressure [111]. To extract meaningful hemodynamic features from this input, Miao et al. [111] propose ResLSTM, a deep learning architecture combining a 50-layer ResNet for morphological feature extraction of key ECG waveform components and a bidirectional LSTM for modeling temporal dependencies across sequential heartbeats. The 50-layer configuration was selected to balance predictive performance and computational efficiency, achieving results comparable to those of deeper 128-layer variants at a reduced training cost. The model was trained and evaluated on the MIMIC-III waveform dataset comprising recordings from 1,711 individuals, partitioned into training (65%), validation (10%), and test (25%) sets, and further validated on an independent ARR database to assess generalizability, with performance reported using RMSE and MAE for both SBP and DBP estimation.

The second modality uses novel triboelectric pulse sensors that capture dual-pulse waveform signals with high sensitivity [112]. Rather than training a generalized model, Yao et al. [112] address inter-individual variability through a personalized partial least squares regression (PLSR) framework, where an individual-specific pulse-to-BP mapping is established by training on two days of data collected from the same subject and evaluated on subsequent days, with each participant contributing 20 data sets. This personalized calibration strategy directly mitigates the variability that limits generalized models, with measurement errors reported consistently low across subjects.

Across both approaches, RMSE and MAE are the standard evaluation metrics, consistent with regression task conventions, and compliance with IEEE and British Hypertension Society (BHS) standards for BP measurement accuracy is increasingly adopted as a benchmark in this domain.

### 6.3. Evaluation Methodology and Generalizability in Cardiovascular Monitoring

Evaluation in cardiovascular monitoring differs from PAR and stress monitoring in two important ways, as summarized in Table 3. For ECG arrhythmia classification, cardiologist consensus annotations provide a clinically meaningful reference standard [110]. For BP estimation, regulatory benchmarks such as IEEE 1708 and British Hypertension Society (BHS) criteria provide quantitative accuracy targets based on measurement error in mmHg. At the same time, data partitioning remains critical: when ICU waveform datasets are split at the record level rather than the patient level, records from the same individual may appear in both training and test sets, leading to overly optimistic estimates of deployment performance [111]. For classification tasks, AUC, ROC-AUC, and per-class F1 are more informative than accuracy alone because arrhythmia datasets are typically class-imbalanced. Future studies should explicitly report patient-level partitioning, clinically grounded reference annotations, and compliance with relevant BP accuracy standards when applicable.

Despite these advances, two challenges remain important. First, many models are trained and evaluated on datasets from specific devices or populations, raising concerns about generalizability across individuals and hardware configurations. Second, the computational demands of deep architectures may conflict with the resource constraints of wearable hardware, motivating future research into domain adaptation, model compression, and efficient on-device inference.

## 7. AI-Driven Personal Exposure Monitoring

**Figure 5:**
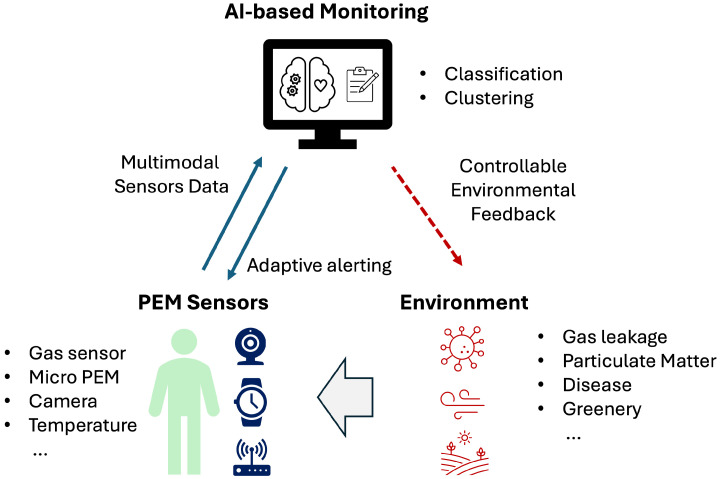
Overview of an AI-based personal exposure monitoring framework. Solid lines indicate the main data flow between multimodal sensors, AI processing, and user interface. Dashed lines represent optional controllable environmental feedback, such as adaptive HVAC, lighting, or ventilation systems.

Wearable technologies have gained significant traction in environmental health monitoring due to their ability to capture ambient conditions at the individual level [113]. Unlike physiological monitoring, which tracks internal biological signals, personal exposure monitoring (PEM) targets external environmental stimuli, such as volatile organic compounds (VOCs), toxic gas emissions, and radiation, that may pose latent health risks before any biological impact is observed [114]. This distinction leads to fundamentally different data characteristics and AI objectives: rather than detecting symptoms or health events that have already occurred, PEM systems prioritize early detection and prediction of hazardous exposure, often relying on signal thresholds, multivariate sensor fusion, and spatiotemporal environmental trends [115,116]. In this sense, PEM systems are inherently anticipatory, expanding the role of wearable technologies from reactive health tracking to proactive risk mitigation [117]. AI algorithms play a critical role in this pipeline by processing complex, high-dimensional environmental inputs to accurately classify pollutants and estimate personal exposure in real time [116,117]. A representative PEM architecture is shown in Figure 5.

### 7.1. Multimodal AI Classification for Virus Detection

Infectious disease surveillance using wearable sensors presents a unique classification challenge: the input is inherently multimodal, combining continuous physiological signals with discrete self-reported symptom data, and the output is a binary infection status label. A fundamental difficulty is that neither modality alone provides sufficient discriminative power, as physiological signals such as resting heart rate and sleep duration reflect only indirect and delayed biological responses to infection, while self-reported symptoms such as cough and fever are subjective and inconsistently recorded. Combined with severe class imbalance due to low infection prevalence in surveillance populations, these constraints demand classifiers that are robust to noisy, heterogeneous inputs and capable of integrating complementary information across modalities.

To address this, XGBoost has been adopted as a suitable classifier given its robustness to heterogeneous inputs and interpretability via feature importance, with the Boruta algorithm applied for feature selection to reduce noise and prevent overfitting, and class weights incorporated into the loss function to mitigate imbalance [66]. Stratified cross-validation with Bayesian hyperparameter optimization ensures stable performance estimates, and the combined multimodal model outperforms both symptom-only and activity-only baselines, achieving a validation AUC of 0.74 with generalizability confirmed on an independent external dataset at AUC 0.75 [66].

### 7.2. AI-Based Classifiers for Air Pollutant Monitoring

Wearable pollutant monitoring poses two interrelated challenges: accurately detecting and classifying hazardous substances from low-cost sensors prone to noise and drift, and contextualizing exposure measurements with respect to user activity. Environmental signals such as VOC concentrations and particulate matter (PM) levels are highly variable due to external conditions and user motion, making robust feature extraction and classifier generalization difficult across real-world deployment scenarios.

For pollutant classification, PCA-based feature extraction combined with a comparative evaluation of multiple classifiers has been shown effective under controlled conditions, with weighted k-NN achieving over 99% classification accuracy and a detection latency of six seconds [118]. For activity-contextualized exposure assessment, a stacked ensemble of heterogeneous classifiers with temporal smoothing has demonstrated strong generalization across sessions, achieving a weighted F1-score of 0.979 (unweighted F1 0.832) under leave-one-session-out cross-validation for bicycling activity detection from PM2.5 and PM10 wearable data [119].

### 7.3. CNN-Based Semantic Scene Segmentation for Greenery Exposure Assessment

Beyond chemical pollutant detection, wearable sensors have been extended to assess perceptual dimensions of environmental exposure, such as contact with urban greenery, which has been associated with mental health and well-being outcomes. The core challenge shifts from chemical signal classification to visual scene understanding, where the input is a continuous stream of egocentric images captured during daily movement and the output is a quantitative estimate of individual-level greenery exposure. CNN-based semantic scene segmentation is well suited to this task, enabling fine-grained, dynamic assessment of visual environmental quality that static geospatial approaches such as Google Street View cannot provide at the individual level [120].

### 7.4. Evaluation Methodology and Generalizability in PEM

Evaluation rigor in PEM varies widely across tasks, as summarized in Table 4. Independent external-dataset validation, as used for influenza detection [66], provides strong evidence of cross-cohort generalization because the model is tested on data collected from different participants and study conditions. Leave-one-session-out validation [119] is also useful for activity-contextualized exposure tasks because it reduces the risk that session-specific correlations dominate the test results. In contrast, evaluation on a single chemical compound under a fixed train/test split [118] is best interpreted as a controlled proof-of-concept rather than as evidence of broad pollutant-detection generalizability. Because PEM systems often involve imbalanced or risk-sensitive outcomes, AUC and F1-based metrics are generally more informative than raw accuracy. Future PEM studies should prioritize external-cohort validation, session- or day-level splits, and multi-compound benchmarks when labeled data are available.

Across PEM domains, a shared limitation is the noisy and context-sensitive nature of environmental input signals, which fluctuate due to external conditions, user motion, and low-cost sensor artifacts [114,116]. Future progress will require robust sensor fusion, adaptive filtering strategies, and evaluation frameworks that better capture the complexity of real-world exposure scenarios.

## 8. AI-Enhanced Sweat Biomarker Monitoring

Sweat has emerged as a rich and noninvasive source of biochemical information, offering continuous insights into physiological states such as hydration, electrolyte balance, and metabolic activity [121,122]. Unlike transient biosignals such as ECG, sweat contains biochemical markers including sodium, potassium, calcium, glucose, and uric acid that reflect underlying health conditions over extended periods. Wearable sensors designed for sweat analysis operate through direct skin contact, often incorporating flexible polymer substrates such as polyethylene terephthalate (PET), polyimide (PI), and polydimethylsiloxane (PDMS) that offer biocompatibility and mechanical stability under real-world conditions [8].

However, sweat biomarkers pose unique analytical challenges: sweat secretion is highly individualized and sporadic, influenced by hydration status, ambient temperature, and physical exertion [123], and analyte concentrations can vary dynamically even over short time intervals. In this context, AI is not only valuable for managing signal noise but is essential for modeling temporal fluctuations and multivariate dependencies that translate irregular sweat data into clinically meaningful insights [124].

### 8.1. Tree-Based Ensemble Methods for Sweat Glucose Monitoring

Sweat glucose monitoring is formulated either as a regression problem, where the output is a continuous interstitial glucose (IG) value, or as a multi-class classification problem, where the output is a discretized glycemic category such as high, standard, or low. The core challenge is that sweat glucose correlates only indirectly with blood glucose, and the input signals—including physiological measurements such as heart rate and skin temperature alongside contextual variables such as time of day and dietary intake—are noisy, multimodal, and subject to significant inter-individual variability.

Tree-based ensemble methods, particularly random forest and XGBoost, have demonstrated strong performance on this task due to their robustness to heterogeneous, noisy inputs and their ability to model nonlinear feature interactions [68]. This study uses Dexcom continuous glucose monitor (CGM) interstitial-fluid readings paired with Empatica E4 wearable signals, so its findings are best interpreted as wearable-mediated interstitial-glucose prediction rather than direct electrochemical sweat sensing. SHAP-based feature importance analysis has further revealed that circadian patterns, captured through time-from-midnight as a contextual predictor, play a significant role in glucose regulation, highlighting the importance of incorporating temporal context alongside raw physiological signals [68]. For direct electrochemical sweat glucose sensing, XGBoost has also been applied to predict current responses from fabrication parameters including carbon nanofiber and carbon nanotube concentrations, achieving an $R^{2}$ of 0.86 and an RMSE of 0.177 under five-fold cross-validation [125]. RMSE and $R^{2}$ are the standard evaluation metrics for regression tasks in this domain, while accuracy and F1-score are adopted for classification.

### 8.2. AI Decoding Pipelines for Sweat pH Monitoring

Sweat pH monitoring using wearable colorimetric sensors poses a distinct signal-decoding challenge: the input is a smartphone-acquired image of a pH-sensitive colorimetric indicator embedded in a wearable substrate, and the output is either a discrete pH class or a continuous pH value. The primary difficulty is that image-based color interpretation is highly sensitive to illumination variability, camera differences, and user handling, making fixed color calibration approaches unreliable under real-world conditions [126,127].

To address this, AI-based decoding pipelines have been adopted to replace static calibration, shifting the burden of accuracy and robustness from sensing chemistry to data-driven inference. For classification tasks, machine learning models trained on colorimetric image features extracted from textile-based, battery-free sensors have demonstrated improved reliability under variable imaging conditions [126]. For regression tasks, machine learning models combined with bilayer hydrogel sensor designs have enabled accurate continuous pH estimation over a wide physiological range [127]. Together, these approaches indicate that AI decoding pipelines can compensate for the limitations of low-cost colorimetric sensors, enabling simpler, wearable-friendly designs without sacrificing practical measurement performance.

### 8.3. AI-Based Interference Mitigation in Sweat Uric Acid and Amino Acid Sensing

Beyond glucose and pH, sweat contains a broader range of biochemically relevant analytes, including uric acid and amino acids, whose accurate quantification under dynamic real-world conditions is challenged by signal interference, sensor fouling, and limited detection specificity. These challenges motivate AI-assisted sensing approaches that go beyond static calibration to enable robust, real-time biomarker inference.

For uric acid sensing, the task is formulated as a regression problem in which the input is an electrochemical signal from a peptide-based composite hydrogel sensor with Au-PdNPs/rGO nanohybrids, and the output is the predicted uric acid concentration in real sweat. An ANN trained on 1,217 experimental datasets with concurrently varying pH (4–9), temperature (5–40 °C), and uric acid (UA) concentrations (0–1000 μM) achieved $R^{2}=0.9989$ on a held-out test set and matched ELISA measurements on real sweat samples [128]. The antifouling peptide hydrogel also reduced signal loss in undiluted sweat to 8.3%, compared with more than 50% for control electrodes. For amino acid monitoring, the task involves predicting tryptophan and tyrosine concentrations from a dual-electrode non-enzymatic sensor, where four explainable features are extracted and sweat pH is incorporated as an auxiliary variable to account for physiological variability [129]. Experimental validation across subjects with and without amino acid supplementation confirms a consistent linear relationship between tryptophan and tyrosine concentrations independent of pH variation [129]. Across both targets, these studies demonstrate that AI-assisted decoding can broaden the scope of reliable wearable biomarker detection beyond what sensing chemistry alone can achieve under dynamic conditions [128,129].

Beyond AI-driven decoding, hardware–material co-design is increasingly important for next-generation sweat biomarker sensors. Two-dimensional (2D) materials such as graphene, transition metal dichalcogenides (e.g., MoS_2_, WSe_2_), black phosphorus, and MXenes offer high carrier mobility, mechanical flexibility, and a large surface-to-volume ratio that benefit wearable electrochemical and optical sensors [130]. For surface-enhanced Raman spectroscopy (SERS) in particular, hybrid substrates combining gold nanoparticles with 2D materials substantially improve signal uniformity and noise characteristics: an AuNP/graphene platform reduces noise by approximately 67% and increases the signal-to-noise ratio by approximately 279% relative to a conventional AuNP substrate, achieving a ten-fold improvement in the limit of detection for protein biomarkers (from $10^{-8}$ to $10^{-9}$ M) [131]. Combining such hardware-level noise management with AI-based decoding pipelines is a promising direction for sweat biomarker sensors that require both chemical specificity and signal stability under dynamic wearing conditions.

### 8.4. Evaluation Methodology and Generalizability in Sweat Biomarker Monitoring

Evaluation in sweat biomarker monitoring (Table 5) requires attention to three issues. First, biochemical ground truth should be specified clearly, such as laboratory reference assays for uric acid [128] or paired CGM interstitial readings for glucose [68]. This distinction is important because not all studies labeled as wearable biomarker monitoring directly measure sweat analytes. Second, the validation setting should be separated into in vitro, artificial-sweat, and on-body real-sweat experiments when applicable, since analytical performance under controlled conditions may not fully reflect biological variability during deployment [125,129]. Third, dataset size, number of participants, number of independent trials, and validation protocol should be reported alongside headline metrics because many sweat-biomarker studies remain relatively small compared with motion- or ECG-based wearable sensing. In addition to accuracy, future studies should report stability, drift, repeated-measure reliability, and agreement with reference assays, using measures such as Bland–Altman analysis or ICC where appropriate.

Across sweat biomarker monitoring domains, AI-driven preprocessing and decoding pipelines help mitigate signal interference, compensate for physiological and environmental fluctuations, and extract biomarker information across diverse targets including glucose, pH, uric acid, and amino acids [121,124]. Future progress will require clearer reporting of reference assays, separate in vitro and on-body validation, and larger real-world studies that evaluate stability and cross-subject reliability.

## 9. Conclusions and Future Directions

### 9.1. Summary of AI-Enhanced Wearable Sensors

**Figure 6:**
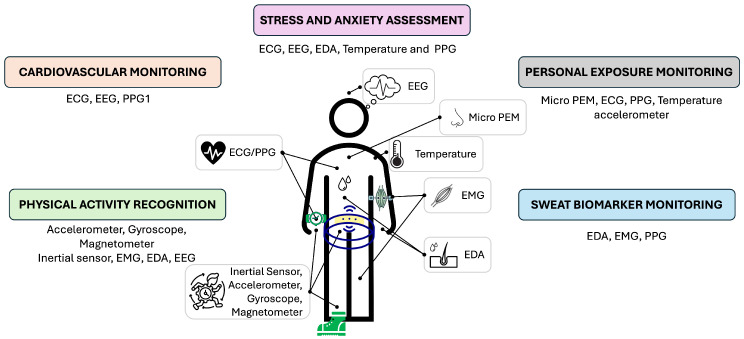
Sensors used for each application of wearable sensors discussed in the review.

This review summarizes the development of AI-enhanced wearable sensors across multiple application domains and sensing modalities. As shown in Figure 6, the reviewed literatures can be organized into five major categories based on task objectives and available sensor streams, including physical activity tracking, cardiovascular monitoring, stress and anxiety monitoring, personal exposure monitoring, and sweat biomarker detection. Across these categories, wearable systems increasingly rely on heterogeneous sensor combinations, reflecting the need to capture complementary physiological, biochemical, and motion-related information for specific tasks.

Table 6 indicates that most reviewed studies predominantly adopt supervised learning paradigms and report strong performance within their target settings. The majority of applications are formulated as classification problems, such as activity recognition, stress detection, rhythm abnormality classification, and disease screening. Traditional machine learning models—including random forests, support vector machines, k-nearest neighbors, logistic regression, decision trees, naive Bayes, and gradient boosted trees—are widely used and often achieve competitive accuracy and F1-scores, demonstrating the continued effectiveness of structured feature representations for many wearable sensing tasks. Deep learning models appear in fewer cases, such as CNN–RNN hybrids for rhythm classification, suggesting that neural architectures are primarily adopted when temporal dynamics and complex signal patterns require higher model expressiveness.

Overall, the evidence from Figure 6 and Table 6 shows that AI- wearable sensors have evolved from single-sensor, signal-level processing toward application-driven systems that increasingly exploit multimodal inputs, while still relying largely on supervised learning evaluated on domain-specific datasets. At the same time, growing attention to data privacy and deployment constraints has motivated interest in privacy-preserving frameworks, such as federated learning, that enable model updates without centralizing sensitive personal data. In parallel, current trends emphasize lightweight and efficient on-device models capable of operating under strict computational and energy budgets. Together, these directions highlight the importance of multimodal fusion, robust learning under dynamic conditions, and efficient model design to support scalable, trustworthy, and real-world deployment of wearable sensing systems.

Table 7 provides a domain-level synthesis across the five application areas reviewed in this paper, highlighting cross-cutting patterns in signal characteristics, dataset scales, dominant ML paradigms, common failure modes, and generalizability challenges.

### 9.2. Key Challenges

AI-enhanced wearable sensors with have revolutionized healthcare, yet several technical and ethical challenges remain in their development and applications.

AI-enhanced wearable systems often transmit sensitive physiological data from body-worn devices to edge or cloud servers for model inference and training. This data pipeline introduces significant privacy risks, as intercepted or improperly protected data may reveal highly sensitive personal information. The increasing reliance on cloud-based AI exacerbates these concerns, emphasizing the urgent need for robust data encryption, secure communication protocols, and privacy-preserving machine learning frameworks to safeguard user data throughout the sensing-to-decision pipeline.

Beyond technical privacy protection, AI-enabled wearable health systems must also satisfy regulatory and ethical requirements before clinical deployment. In the United States, wearable systems that support diagnosis, treatment decisions, or clinical monitoring may fall under software-as-a-medical-device or AI-enabled medical-device regulatory pathways, requiring evidence of safety, effectiveness, transparency, and lifecycle management. In parallel, data governance frameworks such as Health Insurance Portability and Accountability Act (HIPAA) and General Data Protection Regulation (GDPR) require careful handling of identifiable health information, including informed consent, data minimization, secure storage, access control, and restrictions on secondary use. These requirements create practical barriers for clinical translation because model training, validation, and post-deployment monitoring must be designed around privacy protection, auditability, and regulatory compliance from the outset rather than added after prototype development.

A further translational challenge is the gap between research prototypes and clinically validated or commercially deployable wearable AI systems. Many reviewed studies demonstrate promising performance in controlled laboratory settings, retrospective datasets, or small cohorts, but clinical adoption requires prospective validation across diverse populations, devices, and real-world use environments. Major bottlenecks include limited external validation, sensor drift, calibration burden, inter-individual physiological variability, workflow integration with clinical practice, cybersecurity requirements, user adherence, manufacturability, reimbursement pathways, and post-deployment performance monitoring. Therefore, future studies should move beyond proof-of-concept accuracy and report evidence of robustness, usability, calibration burden, clinical workflow compatibility, and long-term reliability.

While many studies validate AI/ML models using techniques such as k-fold cross-validation or leave-one-out validation, these approaches primarily assess statistical performance rather than clinical reliability. Moreover, the blackbox nature of deep learning models limits transparency and interpretability, which undermines trust among healthcare professionals. For wearable-based diagnostic systems to be clinically adopted, explainable AI approaches are required to provide interpretable insights, justify predictions, and support decision-making.

Many AI-enabled wearable systems depend on cloud-based computation, which introduces latency that can delay time-sensitive clinical decisions. Such delays are particularly problematic for applications requiring rapid response, including cardiac monitoring or stress detection. Edge computing and on-device AI inference offer promising solutions by reducing communication latency and enabling real-time analytics. However, achieving low latency while maintaining model accuracy and computational efficiency remains a significant challenge due to the limited resources of wearable platforms.

The performance of AI/ML models is fundamentally dependent on data quality. Wearable sensor data are inherently noisy, incomplete, and subject to motion artifacts and environmental interference. As a result, most studies rely on extensive preprocessing, filtering, and imputation techniques to handle missing or corrupted data. Despite these efforts, variability across users and operating conditions continues to limit model robustness and generalizability, highlighting the need for standardized data acquisition and validation protocols.

Energy efficiency remains one of the most critical limitations of AI-enabled wearable devices. Power consumption is influenced by sensing hardware, sampling rates, data logging frequency, wireless transmission, and display operation. Cloud-dependent AI architectures significantly increase energy demand due to continuous data transmission and bidirectional communication.

Commercially deployed wearable health technologies, such as continuous glucose monitors, ambulatory ECG patches, and smartwatch-based rhythm monitoring, illustrate that successful translation requires not only accurate algorithms but also stable sensing hardware, user adherence, regulatory clearance, clinical workflow integration, reimbursement models, and post-market performance monitoring [5,11,13].

### 9.3. Future Opportunities of AI-Enhanced Wearable Sensors

The future of AI-enhanced wearable sensors lies in the synergistic advancement of sensing hardware, artificial intelligence, and networked healthcare infrastructure. Continued progress in both low-power wearable electronics and efficient AI algorithms is expected to significantly expand the scope of real-time, continuous, and clinically actionable health monitoring. In particular, the development of lightweight edge-AI frameworks will enable on-device inference with minimal latency and power consumption, reducing dependence on cloud computation and supporting time-critical healthcare applications.

One promising direction is the integration of wearable sensors into multimodal healthcare systems. Future wearable platforms will increasingly fuse heterogeneous data streams, including electrophysiological signals (e.g., ECG, PPG), motion data, and biochemical markers, to provide a more comprehensive and context-aware assessment of human health. When combined with seamless interoperability with electronic health records (EHRs) and Internet of Things (IoT) healthcare infrastructures, such multimodal intelligence can enhance clinical decision-making, enable longitudinal health tracking, and support data-driven precision medicine.

Advances in personalized and adaptive healthcare represent another major opportunity. AI-driven wearable systems can move beyond population-level models toward individualized health analytics, enabling personalized diagnosis, treatment recommendations, and risk prediction based on real-time physiological patterns. Self-learning wearable systems that continuously adapt to individual behaviors, lifestyles, and physiological baselines will further improve robustness and clinical relevance, particularly for chronic disease management and preventive care.

From a hardware perspective, emerging technologies such as electronic skin (e-skin) are poised to redefine wearable sensing. E-skin platforms enable ultra-thin, flexible, and skin-conformal sensors that seamlessly interface with the human body, enhancing comfort, signal fidelity, and long-term usability. In parallel, breakthroughs in energy-harvesting technologies, such as triboelectric nanogenerator (TENG)-based systems, offer pathways toward self-powered or energy-autonomous wearables. Such developments are critical for extending device lifetime and enabling maintenance-free, continuous monitoring in real-world settings.

Finally, privacy-preserving and secure AI frameworks will play a pivotal role in the future adoption of AI-enabled wearable systems. As wearable devices generate increasingly sensitive health data, there is growing demand for AI models capable of learning from decentralized data without compromising user privacy. Federated learning, secure edge intelligence, and advanced encryption strategies offer promising solutions. In addition, AI-driven cybersecurity techniques can enhance threat detection, anomaly identification, and resource-efficient security management, ensuring robust protection against evolving cyber risks in wearable–IoT healthcare ecosystems. However, the practical use of federated learning in wearable health systems still faces several deployment barriers. Frequent model updates can introduce communication overhead for low-power wearable devices, and non-identically distributed data across users may reduce the robustness of a shared global model [134,135]. These issues are especially relevant in wearable sensing because users differ in physiology, activity patterns, device placement, sensor quality, sampling rate, and missing-data patterns. Personalized federated learning and federated transfer learning have therefore been proposed to balance global knowledge sharing with user-specific adaptation in wearable healthcare applications [136,137]. Future systems should combine federated optimization with personalization, model compression, missing-modality handling, and robustness evaluation across diverse users and devices.

## Figures and Tables

**Table 1:** Summary of representative AI/ML studies for wearable physical activity recognition (Section 4), comparing task, model, dataset, validation protocol, and reported metric. NR = not reported in the original paper.

Ref.	Task	Model	Dataset/Subjects	Validation	Metric
*Human Activity Recognition (Section 4.1)*					
[84]	3D strain gesture	MLP + fuzzy inference	Single-site lab (n NR)	Holdout (NR)	$R^{2}=0.9286$
[85]	13-class daily HAR	Random forest	Multi-subject (n NR)	10-fold CV (within-cohort)	Acc >94%
[86]	Urban context HAR	RF, DT	18 participants	Within-cohort	Acc 77%
[87]	Maternal activity	Gradient-boosted trees	Pregnant cohort, wrist module	90/10 split	Acc 89%
*Sleep Monitoring (Section 4.2)*					
[83]	Sleep apnea (binary; severity)	MLP, GNB	Real patients	NR	Acc 92%/89%/71%
[88]	3-class workout/awake/sleep	SVM, RF	1 participant, 64 days	Within-subject	F1 >0.96
*Posture and Gesture (Section 4.3)*					
[76]	ASL gesture (27 cls.)	SVM + sensor fusion	12 subjects, 6.4 M instances	Leave-one-out CV	Acc >95%
[81]	Construction posture	GRU	Active construction site	NR	Acc 99.01%
*Rehabilitation and Wellness (Section 4.4)*					
[78]	Stroke rehab motion	Personalized CNN	17 chronic stroke survivors	Within-subj. 5-fold	99.9% personal; 95.8% cross-subj.
[79]	Shoulder exercise (6-class)	Random forest	19 healthy + 17 RCT patients	5-fold (within-cohort)	100%
[89]	Breast cancer screening	CatBoost	Bra-system capacitance	NR	Acc 95%
[90]	Hydration (binary)	LightGBM + multispectral PPG+IMU	19 participants, 103 trials	4-fold (within-cohort)	Acc (binary)
*Motor Disorder Monitoring (Section 4.5)*					
[91]	PD postural instability	Fine-tuned k-NN	PD vs. HC cohort	NR	95.6% (HC vs. PD); 84.2% (ON vs. OFF)
[92]	Tremor (EMG+ACC)	Bagged DT (vs. 6 cls.)	Clinical cohort	NR	Acc 99.6%
[77]	Gait step length	XGBoost	472 subjects (PD, MCI, MS, healthy), 80K+ steps	Cross-cohort	RMSE < 5 cm, ICC = 0.93

**Table 2:** Summary of representative AI/ML studies for wearable stress and anxiety monitoring (Section 5). NR, not reported; MIST, Montreal Imaging Stress Task; TSST, Trier Social Stress Test; GT, ground truth.

Ref.	Task	Model	Dataset/Subjects	Validation	Metric
*Stress-Level Classification (Section 5.1)*					
[96]	Multi-class fatigue/stress (3 sub-problems)	Random forest	Construction workers, insole (plantar pressure + ACC)	10-fold CV (within-cohort)	Acc 86%; P/R/F1 52–84%
[97]	Binary stress vs. calm	k-NN (vs. C4.5, Bayes)	46 participants, smartphone ACC + gyro typing	Within-cohort	Acc 87.56%
[99]	3-class (low/normal/high) stress	Stacked ensemble (RF + GB + meta)	Public Stress-Lysis benchmark, 2001 samples	20% holdout (within-cohort)	Acc 99.5% (benchmark)
[65]	Binary stress vs. non-stress	3-layer stacked LSTM	19 older adults, TSST + salivary cortisol GT	NR	AUC 0.90
[101]	2-level/3-level stress	GB + GA-MI + Bayesian opt. + SHAP	Empatica E4 (BVP + EDA + HRV) under MIST	Cohort split	Acc 98.28%/97.02%
*Mental Health Assessment (Section 5.2)*					
[100]	Stress in MCI	RF, AdaBoost	Older adults with MCI (HRV + EDA)	Cohort split	Acc 85.4/85.3%; AUPRC 0.98/0.97

**Table 3:** Summary of representative AI/ML studies for wearable cardiovascular monitoring (Section 6). NR = not reported.

Ref.	Task	Model	Dataset/Subjects	Validation	Metric
*ECG Monitoring (Section 6.1)*					
[109]	20-class arrhythmia	CNN–RNN hybrid	Wearable ECG (NR)	NR	ROC-AUC 0.9011
[110]	12-class arrhythmia	34-layer deep CNN (pure)	91,232 single-lead ECGs from 53,549 patients (Zio Patch)	Cardiologist consensus	Avg AUC ≈ 0.97; F1 ≈ 0.81
*Blood Pressure Monitoring (Section 6.2)*					
[111]	SBP/DBP regression	ResLSTM (50-layer ResNet + BiLSTM)	MIMIC-III, 1711 individuals, 897,743 records	65/10/25 split + independent ARR database	RMSE, MAE; corr. 0.88/0.71
[112]	Cuffless BP (personalized)	Personalized PLSR	Triboelectric nanopillar pulse sensor; 20 datasets/subj.	Subject-specific train/test	Low MAE per subject

**Table 4:** Summary of representative AI/ML studies for wearable personal exposure monitoring (Section 7). NR = not reported.

Ref.	Task	Model	Dataset/Subjects	Validation	Metric
*Virus Detection (Section 7.1)*					
[66]	Binary influenza detection	XGBoost + Boruta + class weight	953 (HTRI) + 925 (FluStudy2020) participants, Fitbit + symptoms	Stratified *k*-fold + independent HTRI external eval.	Val AUC 0.74; ext AUC 0.75
*Air Pollutant Monitoring (Section 7.2)*					
[118]	Pollutant cls.VOC indoor loc.	Weighted k-NN + PCA	Single compound, controlled indoors	Fixed train/ test split	Acc > 99%; 6 s latency
[119]	Cycling from PM2.5/PM10	Stacked ensemble + temporal smoothing	MicroPEM v3.2b multi-session	Leave-one-session-out CV	Weighted F1 0.979; F1 0.832
*Greenery Exposure (Section 7.3)*					
[120]	Individual greenery exposure	CNN semantic segmentation	Egocentric image stream	Pixel-level eval.	Segmentation metrics

**Table 5:** Summary of representative AI/ML studies for wearable sweat biomarker monitoring (Section 8). NR = not reported.

Ref.	Task	Model	Dataset/Subjects	Validation	Metric
*Sweat Glucose Monitoring (Section 8.1)*					
[68]	Interstitial glucose prediction (cls.& regression)	RF, XGBoost (vs. DT/SVM/LDA/k-NN/GNB/Lasso/Ridge/EN)	Empatica E4 + Dexcom CGM + food log (interstitial, not direct sweat)	Within-cohort	RF $R^{2}=0.84$, RMSE 9.04 mg/dL; SHAP: time-from-midnight
[125]	Electrochemical sweat-glucose current regression	XGBoost (from CNF/CNT concentrations)	In-vitro fabrication-parameter study	5-fold CV	$R^{2}=0.86$; RMSE 0.177
*Sweat pH Monitoring (Section 8.2)*					
[126]	Colorimetric pH & glucose	ML on colorimetric image features (3 algorithms)	Cotton textile sensor + smartphone; standard solutions (pH 4–10; glucose 0.03–1 mM)	In-vitro standard-solution test	Acc ≈ 90%
[127]	Continuous pH + glucose regression	RF + CNN	Bilayer PVA hydrogel (PVA + colorimetric/GOx bottom + PVA-sucrose top) + smartphone app	In-vitro standard solutions (pH 3–9; glucose ≤ 0.5 mM)	$R^{2}\approx 0.99$
*Interference Mitigation: Uric Acid & Amino Acids (Section 8.3)*					
[128]	Uric-acid regression in real sweat (dynamic conditions)	ANN (3 inputs: T, pH, and current–time response) 1 hidden layer, 12 neurons, Adam)	Peptide composite hydrogel + Au-PdNPs/rGO; 1217 datasets across pH 4–9, T 5–40 °C, UA 0–1000 μM	85/15 train+val/test split; ELISA reference	$R^{2}=0.9989$; antifouling 8.3% loss in undiluted sweat
[129]	Trp + Tyr + sweat pH (joint prediction)	Two-electrode non-enzymatic + ML (4 explainable features)	Subjects with/without amino-acid supplementation during cycling trials	Cross-subject (supp. vs. non-supp.)	Trp/Tyr linearity preserved across pH; quantitative metrics NR in abstract

**Table 6:** Summary of AI and ML models used in wearable sensors by application. Abbreviations used in this table: GSR, galvanic skin response; HR, heart rate; RR, respiration rate; BVP, blood volume pulse; NN, neural network; SVM, support vector machine; LR, logistic regression; DT, decision tree; BN, Bayes network; GBT, gradient boosted trees; RF, random forest.

Application	Input	Output Task	Model	Evaluation
Activity classification [86]	Fitness tracker data, including heart rate, step count, energy expenditure, and PM2.5 exposure	Multiclass classification of walking, sleeping, cooking, and exercising	RF	77% accuracy
Workplace activity classification [80]	GSR, HR, RR, BVP, and skin temperature	Binary and multiclass classification of productive versus nonproductive activities, including oiling and cleaning	NN, k-NN, SVM, LR	88% accuracy using NN
Sign language recognition [76]	Flex sensors, pressure sensors, and 3-axis IMU	Twenty-seven-class ASL gesture classification	SVM	>95% accuracy
Keystroke-based stress detection [97]	Accelerometer, gyroscope, and keystroke patterns	Binary classification of relaxed versus stressed states	DT, BN, k-NN	87.56% accuracy using k-NN
ECG emotion stress fusion [98]	ECG and facial emotion features	Binary stress detection	RF	83.33% accuracy
ECG rhythm abnormality classification [109]	Filtered ECG signals from a wearable sensor	Twenty-class rhythm classification	CNN + RNN hybrid	ROC-AUC of 0.9011
Influenza detection [66]	Wearable data, including heart rate and sleep, combined with symptom reports	Binary influenza detection	XGBoost	Combined AUC of 0.74 and external AUC of 0.75
Cycling activity detection [119]	Time-series PM data collected using RTI MicroPEM	Binary classification of cycling versus noncycling	Logistic regression, k-NN, GBT, ensemble	F1 score of 0.979 using ensemble learning
Sweat-based glucose sensing [125]	Sensor fabrication parameters, such as nanofiber concentration	Regression for current response prediction	XGBoost	$R^{2}=0.86$ and RMSE = 0.177
Hunger detection [132]	Respiration belt, EDA, ECG, and EMG	Binary classification of hunger state	RF and deep learning models	RF achieved 93.43% accuracy and 87.86% F1 score
COVID-19 screening [133]	Wearable physiological data	Binary classification of COVID-19 risk	SVM	F1 score of 96.64% and inference time of 0.1521 s

**Table 7:** Cross-domain comparison of wearable AI systems across the five application areas reviewed in this paper.

Domain	Signal Characteristics	Dataset Scale	Dominant ML Paradigms	Common Failure Modes	Generalizability Challenges
PAR (Section 4)	IMU + strain, 50–200 Hz; multivariate time series	10–500 subjects; minutes to hours per subject	RF, GBT, CNN/RNN; personalized models common	Motion artifacts; sensor placement variability; activity ambiguity	Cross-subject generalization; sensor placement
Stress (Section 5)	HRV, EDA, BVP, skin temperature; multimodal physiological + behavioral	10–100 subjects; structured stress-induction sessions	Ensemble (RF, GBT); stacked LSTM	Subjective labels; class imbalance; baseline drift	Population variability; label noise; protocol heterogeneity
Cardio (Section 6)	Single-lead ECG, PPG; ≥200 Hz; large public datasets	1000–90,000+ records (ICU, ambulatory)	Deep CNN, CNN–RNN hybrids, ResLSTM	Class imbalance for rare arrhythmias; patient-vs-record splits	Patient-level partitioning; device-specific calibration
PEM (Section 7)	VOC, PM, environmental + biometric; low-frequency multivariate	10–1000 participants; multi-session	XGBoost, weighted k-NN; stacked ensembles; CNN segmentation	Sensor drift; contextual variability; low SNR	Cross-environment generalization; multi-pollutant benchmarks
Sweat (Section 8)	Electrochemical or colorimetric; sparse, intermittent	100–1200+ measurements; often in vitro	ANN, RF, XGBoost; SHAP for interpretation	Biofouling; individual sweat-rate variation; indirect biomarker mapping	Reference-assay agreement; on-body vs. in vitro performance

## Data Availability

No new data were created or analyzed in this study. Data sharing is not applicable to this article.
